# From mitochondrial oxidative stress to neuroinflammation: integrated proteomic and transcriptomic profiling reveals the role of the ROS/TXNIP/NLRP3 signaling pathway in anxious depression

**DOI:** 10.3389/fimmu.2026.1803956

**Published:** 2026-05-19

**Authors:** Lingchang Shi, Jingya Wei, Ronglin Chen, Yang Liu, Manshu Zou, Jie Luo, Kexin He, Shuangjuan Liu, Yuhong Wang, Pan Meng, Hongqing Zhao

**Affiliations:** 1 School of Medicine, Hunan University of Chinese Medicine, Changsha, Hunan, China; 2 Academy of Chinese Medical Sciences/Science & Technology Innovation Center, Hunan University of Chinese Medicine, Changsha, Hunan, China; 3 Hunan Key Laboratory of Traditional Chinese Medicine Prevention & Treatment of Depressive Diseases, Changsha, Hunan, China; 4 School of Pharmacy, Hunan University of Chinese Medicine, Changsha, Hunan, China

**Keywords:** anxious depression, mitochondrial oxidative stress, Mito-TEMPO, neuroinflammation, ROS/TXNIP/NLRP3

## Abstract

**Background:**

Anxious depression is a prevalent affective disorder characterized by typical depressive symptoms accompanied by persistent anxiety. Increasing evidence suggests that mitochondrial oxidative stress and neuroinflammation contribute to its onset and progression; however, the precise mechanisms linking these processes remain elusive.

**Methods:**

A rat model of anxious depression was established via chronic restraint stress combined with corticosterone administration. Integrated proteomic and transcriptomic analyses were performed to profile differentially expressed molecules in the hippocampus, with key signaling pathways validated by Western blot and qPCR. A Mito-TEMPO intervention group was introduced to evaluate the role of mitochondrial oxidative stress, involving behavioral tests, mitochondrial ultrastructure and reactive oxygen species (ROS) detection, and analysis of autophagy-, inflammation-, and apoptosis-related markers. Additionally, *in vitro* experiments were conducted using TXNIP siRNA knockdown in BV2 microglia to further verify the role of TXNIP.

**Results:**

Proteomic and transcriptomic profiling identified dysregulation of inflammatory-, synaptic-, and mitophagy-related pathways in the hippocampus of model rats, with prominent upregulation of TXNIP and activation of the NLRP3 inflammasome. Model rats exhibited excessive mitochondrial ROS (mtROS) accumulation, suppressed PINK1/Parkin-mediated mitophagy, neuronal injury, and anxiety- and depression-like behaviors. Mito-TEMPO intervention alleviated these pathological changes by scavenging mtROS, restoring mitophagy, inhibiting TXNIP/NLRP3 inflammasome activation, and reducing neuronal apoptosis. *In vitro*, TXNIP knockdown in BV2 microglia attenuated LPS-induced NLRP3 inflammasome activation and pro-inflammatory cytokine secretion, thereby protecting HT22 neurons from inflammatory injury.

**Conclusion:**

Our findings suggest that the ROS/TXNIP/NLRP3 signaling axis mediates the crosstalk between mitochondrial oxidative stress and neuroinflammation, thereby contributing to the pathogenesis of anxious depression.

## 1 Introduction

Anxious depression, a prevalent and treatment-resistant subtype of major depressive disorder (MDD), is characterized by the co-occurrence of persistent anxiety and depressive symptoms; it affects approximately 40% of individuals with depression (1, 2). Compared with non-anxious depression, this subtype is associated with poorer clinical outcomes, including higher relapse rates and increased suicidality (3). Despite the significant clinical implications of anxious depression, the underlying neurobiological mechanisms remain poorly understood, impeding the development of targeted therapeutic approaches. Increasing evidence highlights neuroinflammation and oxidative stress as key pathophysiological processes in depression (4–6). The hippocampus, a critical brain region involved in emotional regulation and stress responses, is especially vulnerable to stress-induced damage. The happocampus is strongly linked to several depressive-like behaviors, including anhedonia, behavioral despair, and cognitive impairments (7). Moreover, neuroinflammation and microglial activation within the hippocampus further exacerbate neuronal damage and impair memory (8).

Mitochondrial dysfunction is increasingly recognized as an important pathophysiological feature in stress-related emotional disorders. By disrupting redox homeostasis, it promotes the pathological overproduction of mitochondrial reactive oxygen species (mtROS), which serve as a pivotal mediator of subsequent neuronal injury and neuroinflammatory activation (9, 10). This surge in mtROS not only acts as a toxic byproduct of impaired oxidative phosphorylation, causing molecular damage to mtDNA, lipids, and proteins, but also is associated with the activation of the NOD-like receptor protein 3 (NLRP3) inflammasome (11, 12). Upon NLRP3 activation, caspase-1 mediates the maturation and release of pro-inflammatory cytokines, including IL-1β and IL-18, resulting in synaptic deficits, impaired neurogenesis, and the emergence of depressive-like behaviors (8). Furthermore, the activation process impairs the clearance of damaged mitochondria, leading to the persistent generation of mtROS. This situation creates a self-reinforcing cycle of mitochondrial damage-autophagic dysfunction-exacerbated oxidative stress (13). This cycle is intricately linked with inflammatory pathways, and is considered to contribute to the sustained and amplified neuroinflammatory state (14).

At present, research in the area of neurological disorders remains fragmented and is largely confined to descriptive correlational observations and conventional approaches such as behavioral pharmacology and single-target assays (15–17). Anxious depression does not arise from a single molecular target or pathway; instead, it reflects complex and interrelated multilevel pathophysiological disturbances. A critical knowledge gap persists at the molecular level spanning from gene expression regulation to protein function, particularly in the systematic identification and validation of mechanistic links between oxidative stress and neuroinflammation. This situation underscores the need to employ integrated transcriptomic and proteomic approaches to explore the mechanisms underlying complex neuropsychiatric disorders. Given that depression involves multifaceted dysregulation across genomic, proteomic, and metabolic networks, such approaches provide a more comprehensive, systems-level perspective, and have contributed to recent advances in the field of neuropsychiatric research (18–20).

Here, we used an integrated proteomic and transcriptomic strategy to explore potential pathological mechanisms underlying anxiety- and depression-like behaviors in a rat model induced by chronic restraint stress combined with corticosterone (CRS/CORT) administration. Through targeted intervention using the mitochondrial-specific antioxidant Mito-TEMPO, we further examined the relationship between oxidative stress and neuroinflammation in this disease context. Our findings provide evidence supporting the involvement of the ROS/TXNIP/NLRP3 signaling pathway in stress-related emotional dysfunction.

## 2 Materials and methods

### 2.1 Animals

Male Sprague-Dawley (SD) rats (weighing 200–220 g) were purchased from Hunan Slac Jingda Laboratory Animal (Changsha, China; license No. SCXK (Xiang) 2019-0004). All rats were housed in an enclosure subject to a standard 12-h light/da k cycle (lights on at 6:00 a.m.) with a constant temperature of 22 ± 2 °C and a relative humidity of 60 ± 10%. The rats were housed in groups of three per cage with free access to food pellets and drinking water. All of the experimental procedures were conducted in compliance with the Guide for the Care and Use of Laboratory Animals of the National Institutes of Health and were approved by the Animal Experiment Ethics Committee of Hunan University of Chinese Medicine (Approval No.: LLBH202306280001).

### 2.2 Experimental design and pharmacological intervention

This study consisted of two experimental phases: a discovery phase for multi-omics screening and an intervention phase for functional validation.

**Figure 1:**
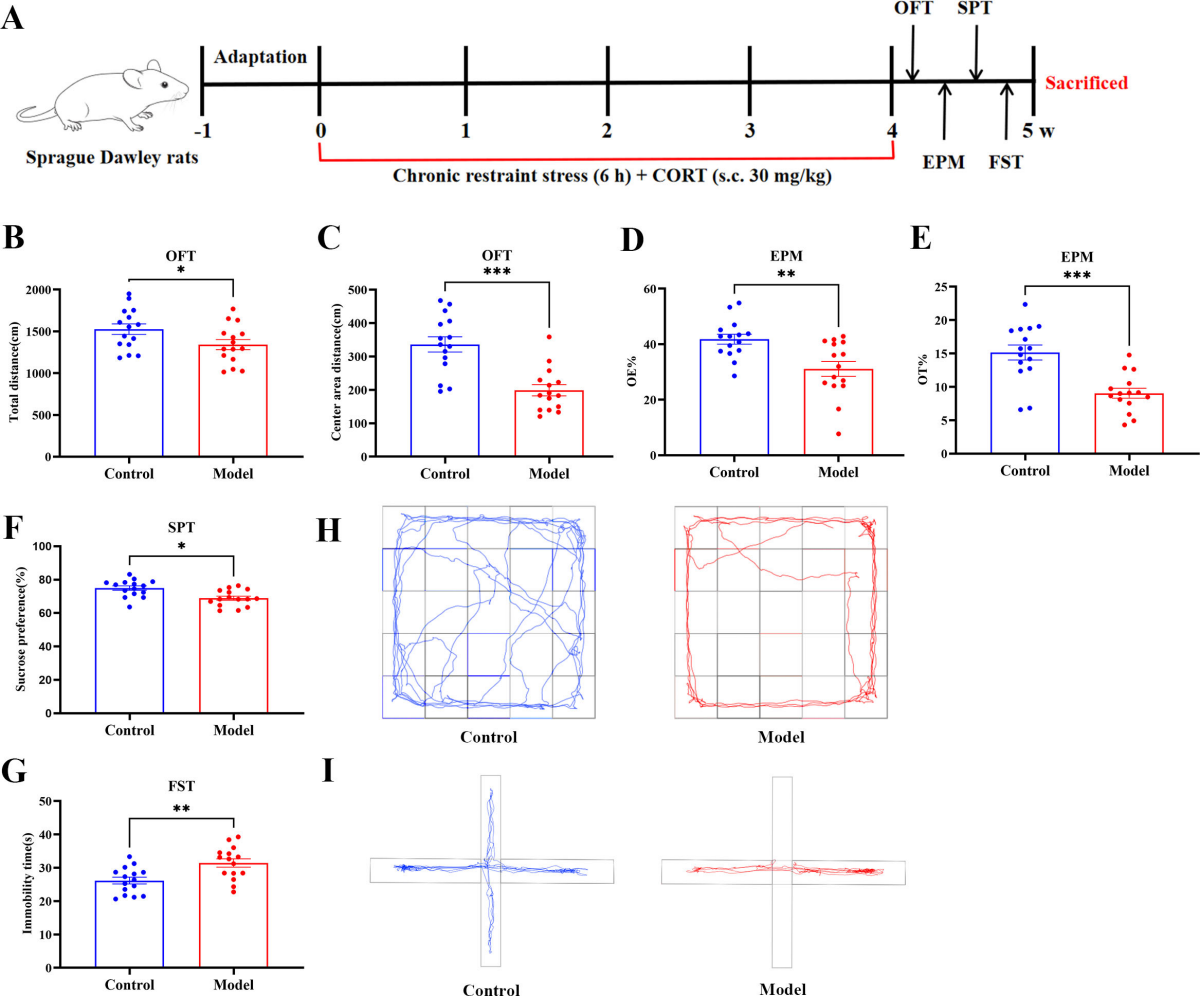
CRS/CORT induces anxiety- and depression-like behaviors. **(A)** The experimental timeline of interventions and behavioral tests. **(B)** The total distance moved by rats in the OFT. **(C)** The distance traveled by rats in the center area in the OFT. **(D)** The open arm entries (OE%) in the EPM. **(E)** The open arm time (OT%) in the EPM. **(F)** Immobility time in the FST. **(G)** Sucrose preference in the SPT. **(H)** Representative movement traces of rats in the OFT. **(I)** Representative movement traces of rats in the EPM. All data are presented as mean ± SEM (*n* = 15 per group). Student’s *t*-test was used for statistical analysis. ^*^p < 0.05, ^**^p < 0.01, ^***^p < 0.001.

Phase 1 (Discovery phase): After acclimatization, all rats were subjected to open field test (OFT) and sucrose preference test (SPT) for baseline behavioral measurements. Rats with significantly deviant behavioral performance were excluded, and the remaining rats were randomly divided into two groups (n = 15 per group): Control and Model. The Model group was subjected to CRS/CORT administration to induce anxiety- and depression-like behaviors (21, 22). Specifically, rats were restrained in well-ventilated tubes for 6 h daily (9:00 a.m. to 3:00 p.m.) for 21 consecutive days, followed by subcutaneous injection of CORT (30 mg/kg). Control rats received gentle handling and vehicle treatment. Behavioral tests were conducted after the modeling period, and hippocampal tissues were collected for transcriptomic and proteomic analyses. The experimental timeline is shown in **Figure 1A**.

**Figure 2:**
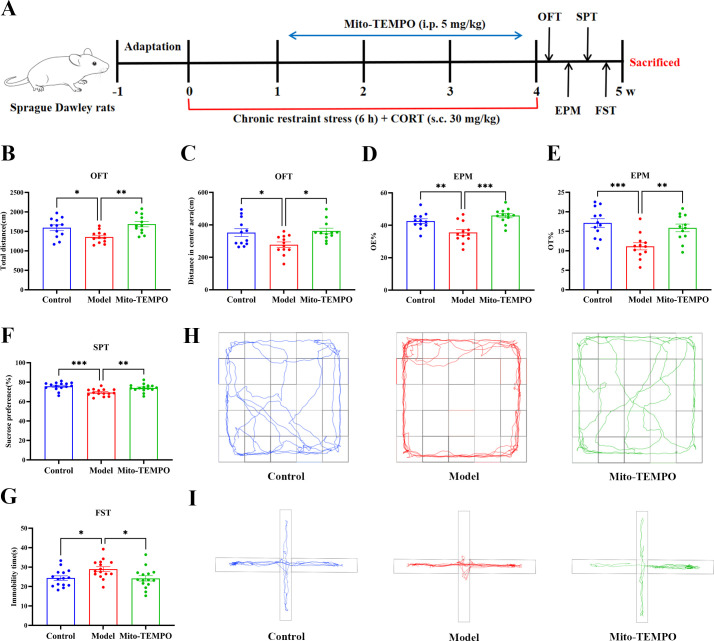
Mito-TEMPO alleviates anxiety- and depression-like behaviors. **(A)** The experimental timeline of interventions and behavioral tests. **(B)** The total distance moved by rats in the OFT. **(C)** The distance traveled by rats in the center area in the OFT. **(D)** The OE% in the EPM. **(E)** The OT% in the EPM. **(F)** Immobility time in the FST as a measure of depression-like behavior. **(G)** Sucrose preference in the SPT. **(H)** Representative movement traces of rats in the OFT. **(I)** Representative movement traces of rats in the EPM. All data are presented as mean ± SEM (*n* = 15 per group). One-way ANOVA with Tukey’s *post hoc* test was used for statistical analysis. ^*^p < 0.05, ^**^p < 0.01, ^***^p < 0.001.

Phase 2 (Intervention phase): A separate cohort of rats was randomly assigned to three groups (n = 15 per group): Control, Model, and Mito-TEMPO. The Model and Mito-TEMPO groups underwent the same CRS/CORT procedure as described above. The Mito-TEMPO group received intraperitoneal injection of Mito-TEMPO (5 mg/kg) 30 min before each restraint session during the 21-day modeling period (23), while Control and Model groups received an equal volume of saline. Behavioral tests were performed 24 h after the final stress session, followed by tissue collection for further analysis. The experimental timeline is shown in **Figure 2A**.

To further investigate the causal role of thioredoxin-interacting protein (TXNIP) in microglial inflammasome activation and its impact on neuronal injury, *in vitro* experiments using BV2 and HT22 cells were conducted as described below.

### 2.3 Behavioral tests

#### 2.3.1 Open field test

We performed the OFT as previously described to evaluate locomotion and anxiety-like behavior in rats (24, 25). The open field apparatus was a 100 × 100 × 40 cm blue box divided into 25 squares at the bottom, with 9 center squares designated as the central area and 16 peripheral squares designated as the peripheral area. The rats were placed in the center of the apparatus and allowed to explore freely for 5 minutes under soft lighting and quiet conditions. The total distance and time spent in the central area were recorded by video tracking software (Track, China).

#### 2.3.2 Elevated plus maze

We employed the elevated plus maze (EPM) as previously described to evaluate anxiety-like behavior in rats (26). The maze is a cross-shaped apparatus 70 cm above the floor that consists of two open arms (30 × 5 cm) and two closed arms (30 × 5 cm) symmetrically extending from a central platform (10 × 10 cm). A video-tracking system was used to record the time that the animals spent in the closed arms (closed-arm time; CT) and the time that the animals spent in the open arms (open-arm time; OT), and the frequency of open-arm entries (OE) and closed-arm entries (CE). The percentages of open-arm entries (OE%) and open-arm time (OT%) were calculated as follows: OE% = [OE/(OE + CE)] × 100; OT% = [OT/(OT + CT)] × 100.

#### 2.3.3 Sucrose preference test

We performed the SPT as previously described to evaluate depressive-like behavior in rats (24, 26). The rats were habituated to two bottles of 1% sucrose solution for 24 h. Following this habituation, the animals were deprived of food and water for 12 h before the final test. On the test day, each rat received two pre-weighed bottles containing water and sucrose solution at 1% (w/v) 2 h; the positions of the two bottles were switched in the middle of testing. The sucrose preference ratio was calculated by the following formula: sucrose preference% = [sucrose consumption (g)/(sucrose consumption (g) + water consumption (g))] × 100.

#### 2.3.4 Forced swim test

We used the forced swim test (FST) as previously described to evaluate depressive-like behavior in rats (24, 25). During the experiment, the rats were placed individually into a transparent cylinder (50 cm in height, 30 cm in diameter) that was filled with water at a temperature of 24 ± 1 °C to a depth of 35 cm. All of the rats were forced to swim for 6 min, and the animals’ immobility time was recorded over the last 4 min. The immobility time was defined as rats floating upright and making minimal movements to keep their heads above the surface of the water.

### 2.4 Sample collection

After behavioral testing, rats were anesthetized with sodium pentobarbital (50 mg/kg, i.p.), and blood was collected via abdominal aorta puncture. Serum or plasma was obtained by centrifugation and stored at -80°C. Rats were then perfused with pre-cooled saline followed by 4% paraformaldehyde. Brains were rapidly removed, and hippocampi were dissected for subsequent analyses: tissues for omics were rinsed and snap-frozen at -80°C; samples for histopathology were post-fixed, dehydrated, embedded, and sectioned; and samples for electron microscopy were fixed in glutaraldehyde–paraformaldehyde at 4°C.

### 2.5 Hippocampus slice preparation and mitoSOX

We measured the mtROS levels using the MitoSOX Red Mitochondrial Superoxide Indicator (RM02822, Abclonal, China). Rats were anesthetized and sacrificed with pentobarbital and the brain was removed rapidly and placed into ice-cold Hanks Balanced Salt Solution (PB180323, Pricella, China). Coronal slices 250 μm wide containing the hippocampus were cut using a vibratome (Leica, VT1200s, Germany). The slices were incubated with 5 μmol/L MitoSOX Red at 37 °C in the dark for 15 min and then washed three times with PBS. Next, the slices were incubated with DAPI working solution (C0060, Solarbio, China) at 37 °C in the dark for 15 min and then washed three times with PBS. The mtROS intensity was assessed via laser confocal microscopy (A1R HD25, Nikon, Japan).

### 2.6 Tandem mass tag analysis of hippocampus proteomics

The proteomic analysis service was conducted by Guangzhou Weiyu Zhihe Technology Co., Ltd. (Guangzhou, China). Rats used for the omics analyses were randomly selected from each group after the completion of the behavioral testing. Total protein was extracted from the hippocampus. After quantification, 30 μL of protein extract from each sample was subjected to enzymatic digestion. Next, a 100 μg aliquot of the resulting peptide mixture from each sample was labeled using Tandem Mass Tag (TMT) reagent according to the manufacturer’s instructions (Thermo Fisher Scientific, USA). The six hippocampal samples were labeled as follows: Control-1, Control-2, Control-3, Model-1, Model-2, and Model-3 were labeled TMT-127C, TMT-128C, TMT-129C, TMT-130C, TMT-131C and TMT-132C, respectively. A high-pH RP spin column was used for fractionation after the labeled peptides of each group were mixed in equal amounts. The samples were separated by chromatography (Easy nLC; Thermo Fisher Scientific) and analyzed by mass spectrometry (Thermo Fisher Scientific). Proteins were identified and quantified from the raw data using Mascot 2.2 (Matrix Science, USA) and Proteome Discoverer 1.4 (Thermo Fisher Scientific). Proteins with a fold change >1.2 or <0.83 and a *p*-value < 0.05 were defined as differentially expressed proteins (DEPs) (27, 28).

### 2.7 RNA-seq and transcriptomics of hippocampus

The RNA-sequencing service was provided by Guangzhou Weiyu Zhihe Technology Co., Ltd. After total RNA was fragmented into short fragments and mRNA was enriched using oligo (dT) magnetic beads, the first strand of cDNA was synthesized. Double-stranded cDNA was purified and enriched by PCR amplification. The clustering of the index-coded samples was performed on a cBot cluster generation system using HiSeq PE Cluster Kit v4-cBot-HS (Illumina, USA) according to the manufacturer’s instructions. After clusters were generated, the libraries were sequenced on an Illumina platform and 150-bp paired-end reads were generated. Altered (upregulated or downregulated) expression of genes was represented by the log2-transformed fold change (log2FC), calculated as log2FC = log2(B) - log2(A), where A and B represent the gene expression values in the two groups being compared.

### 2.8 Functional and network analysis

We performed functional annotation was performed using the DAVID database (https://david.ncifcrf.gov/) for Gene Ontology (GO) and KEGG pathway enrichment analyses. A KEGG pathway was considered significantly enriched at a *p*-value < 0.05 and false discovery rate (FDR) < 0.05. A Protein-Protein Interaction (PPI) network was constructed using the STRING database (confidence score > 0.4) and visualized in Cytoscape. Its functional modules were annotated based on STRING-derived biological processes. To ensure consistency, all protein identifiers were uniformly converted to standard Gene IDs using the UniProt and Ensembl databases.

### 2.9 Hematoxylin & eosin staining, Nissl staining and TUNEL staining

Rat brain tissue fixed in paraformaldehyde was processed for paraffin embedding and sectioned into 5-μm slices. We performed staining with hematoxylin & eosin (HE) using the HE kit (G1120, Solarbio, China). All steps were performed in strict accordance with the manufacturer’s protocols. We examined. We examined the pathological changes in the hippocampal tissue in each group using a slide scanner (Pannoramic MIDI, 3DHISTECH, Hungary). We performed Nissl staining was performed according to the manufacturer’s instructions using a commercial kit (G1432, Solarbio) to detect changes in Nissl bodies within the hippocampus. Apoptosis in the hippocampus was detected by TUNEL staining using a kit according to the manufacturer’s instructions (11684817919, Roche, Switzerland).

### 2.10 Immunofluorescence staining

Paraffin-embedded brain sections (5-6 µm thick) were deparaffinized, rehydrated, and subjected to antigen retrieval using citric acid buffer (pH 6.0) via microwave heating. After blocking with 10% goat serum, the sections were incubated overnight with primary antibodies, followed by secondary antibody incubation for 1 hour. The nuclei were stained with DAPI (C0060, Solarbio), and the sections were mounted with anti-fade medium for fluorescence microscopy.

### 2.11 Transmission electron microscopy

Hippocampal tissue samples from each group were fixed in 1% osmium tetroxide, stained with uranyl acetate aqueous solution, and subsequently dehydrated and embedded in epoxy resin. Ultrathin sections were then stained with lead citrate and examined using transmission electron microscopy (TEM) with a Hitachi HT7800 (Japan) microscope to assess the morphological features of mitochondria and autophagosomes.

### 2.12 Quantitative real-time polymerase chain reaction analysis

We isolated total RNA from hippocampal tissues using TRIzol reagent (Invitrogen, USA) according to the manufacturer’s instructions. NovoScript Plus All-in-one 1^st^ Strand cDNA Synthesis SuperMix Kit (E047-01B, Novoprotein, China) was used to remove gDNA and reverse transcribe RNA into cDNA. The cDNA was amplified using the NovoStart SYBR quantitative real-time polymerase chain reaction (qPCR) SuperMix Plus (E096-01A, Novoprotein) kit and run in the CFX96 Real-Time System (Bio-Rad, USA) according to standard methods. The sequences for primers are listed in **Supplementary Table 1**.

### 2.13 Enzyme-linked immunosorbent assay

The concentrations of IL-1β, IL-18, IL-6, TNF-α, and BDNF in the hippocampus were quantified using commercial enzyme-linked immunosorbent assay (ELISA) kits, and the absorbance was measured with a Multi-Mode Microplate Reader (Varioskan Flash, Thermo Fisher Scientific). All of the measurements were performed according to the manufacturer’s protocols.

### 2.14 Western blot analysis

We extracted protein samples were extracted from the hippocampus using 500 μl of RIPA buffer supplemented with a protease inhibitor cocktail (Complete, Roche, Germany) on ice. BCA kit (P0012, Beyotime, China) was used to determine the protein concentrations. Equal amounts of protein were separated by SDS-PAGE and then transferred onto a nitrocellulose membrane using a Mini Transfer System (Bio-Rad, USA). After blocking with 5% nonfat milk, membranes were incubated overnight at 4°C with primary antibodies. Details of primary antibodies, including sources and dilution ratios, are provided in **Supplementary Table 2**. Membranes were then incubated with HRP-conjugated secondary antibodies (1:8000, Proteintech, China), followed by incubation with an ECL substrate (WBKLS0500, Merck Millipore, Germany). Signals were detected using an imaging system (Bio-Rad, USA).

### 2.15 *In vitro* validation of TXNIP-mediated neuroinflammation

BV2 microglial cells were obtained from ProCell (Wuhan, China) and cultured in DMEM (Gibco, USA) supplemented with 10% fetal bovine serum (FBS) and 1% penicillin-streptomycin at 37°C in a humidified atmosphere containing 5% CO_2_. For TXNIP knockdown, cells were transfected with TXNIP-specific siRNA targeting mouse TXNIP or negative control siRNA (designed and synthesized by BrainCase Biotech Co., Ltd., Shenzhen, China) using Lipofectamine 3000 (Invitrogen, USA) according to the manufacturer’s instructions. The final siRNA concentration was 100 nM. The siRNA sequences are provided in **Supplementary Table 1**. After 6 h of transfection, the medium was replaced with fresh complete medium, and cells were incubated for an additional 18 h. The efficiency of TXNIP knockdown was confirmed by qPCR and Western blot analysis.

After transfection for 24 h, BV2 cells were stimulated with lipopolysaccharide (LPS, Sigma-Aldrich, USA) at a concentration of 100 ng/mL for 24 h to induce inflammatory activation (29). Cells were divided into four groups: (1) Control group: untreated cells; (2) si-TXNIP group: cells transfected with TXNIP siRNA; (3) Model group: cells treated with LPS; (4) Model + si-TXNIP group: cells transfected with TXNIP siRNA followed by LPS stimulation. After treatment, culture supernatants were collected for the measurement of inflammatory cytokines using ELISA kits. Cells were harvested for protein extraction, and Western blot analysis was performed to detect the expression of TXNIP, NLRP3, ASC, and caspase-1. Conditioned medium (CM) was prepared from BV2 cells after the above treatments (30, 31). Briefly, BV2 cells were washed twice with PBS and then incubated in serum-free DMEM for 24 h. The supernatants were collected and centrifuged at 1,500 rpm for 5 min at 4°C to remove cell debris, followed by filtration through a 0.22 μm filter. The resulting conditioned medium was used immediately or stored at -80°C and thawed only once prior to use.

HT22 hippocampal neuronal cells were cultured in DMEM supplemented with 10% FBS under standard conditions. Cells were divided into three groups: (1) CM-Control group: treated with conditioned medium from control BV2 cells; (2) CM-Model group: treated with conditioned medium from LPS-treated BV2 cells; (3) CM-si-TXNIP group: treated with conditioned medium from TXNIP-silenced BV2 cells exposed to LPS. HT22 cells were incubated with the respective conditioned media for 24 h before further analysis. Cell viability was assessed using a CCK-8 assay according to the manufacturer’s instructions (32). For synaptic protein analysis, immunofluorescence staining was performed to detect SYN1 and PSD95 expression. For apoptosis analysis, cells were lysed and subjected to Western blot to detect Bax, Bcl-2, and cleaved caspase-3 expression. procedures were performed as described above. All related experimental procedures were performed as described above or according to previously reported methods.

### 2.16 Statistical analysis

The intensities of the immunofluorescence and the Western blot bands were quantified using ImageJ software. Data were analyzed with GraphPad Prism 9.0 and expressed as mean ± standard error of the mean (SEM). Normality was assessed using the Shapiro-Wilk test, and homogeneity of variance was evaluated using the F-test for two-group comparisons or the Brown-Forsythe test for multi-group comparisons, ensuring that the assumptions for parametric tests were satisfied. For comparisons between two groups, Student’s t-test was used, with homogeneity of variance assessed by the F-test. For comparisons involving multiple groups, one-way or two-way analysis of variance (ANOVA) was performed, followed by Tukey’s *post hoc* test. Normality was assumed based on the robustness of parametric tests. A *p*-value < 0.05 was considered to be statistically significant.

## 3 Results

### 3.1 CRS/CORT induces anxiety- and depression-like behaviors

We conducted a comprehensive series of behavioral tests to systematically validate anxiety- and depression-like behaviors in the rat model. In the OFT, the animals in Model group exhibited a significant decrease in total distance traveled, along with reduced entries into and distance moved in the central zone (**Figures 1B, C, H**). These findings are indicative of prominent anxiety-like behavior and locomotor inhibition (**Figures 1B, C, H**). Heightened anxiety was further corroborated in the EPM, as shown by substantial declines in both the percentage of OE% and OT% (**Figures 1D, E, I**). The SPT revealed marked anhedonia, reflected by a reduced sucrose preference ratio (**Figure 1F**). Concurrently, the FST indicated enhanced behavioral despair, evidenced by increased immobility time (**Figure 1G**). Collectively, these behavioral outcomes confirm that CRS/CORT administration effectively induces anxiety- and depression-like behaviors in rats.

### 3.2 CRS/CORT disrupts hippocampal neurotransmitters and triggers neuronal apoptosis

**Figure 3:**
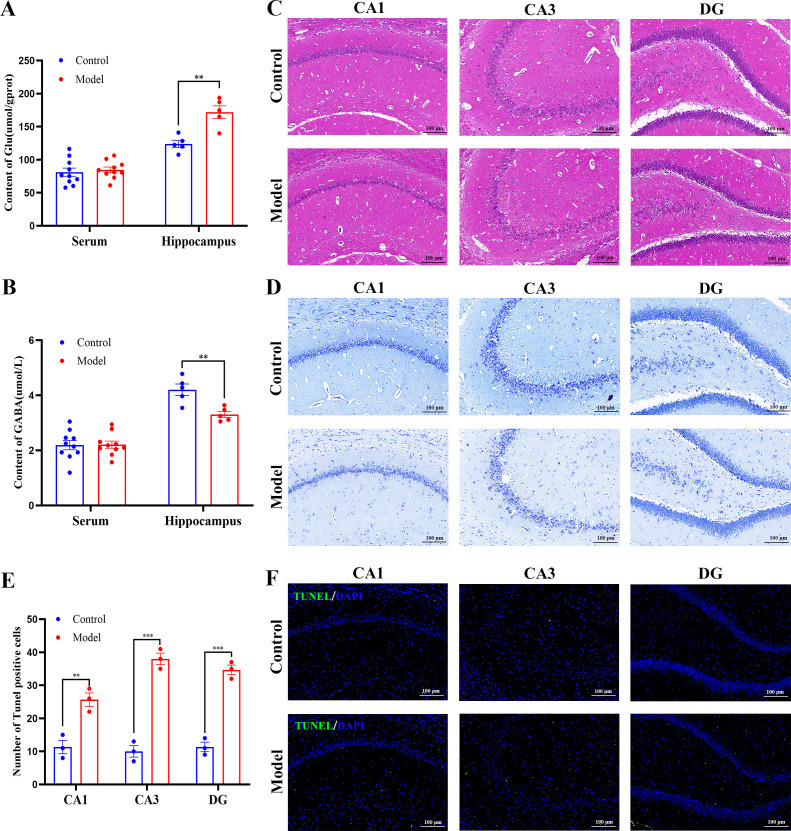
CRS/CORT disrupts hippocampal neurotransmitters and triggers neuronal apoptosis. **(A)** Glu content in serum (*n* = 10) and whole hippocampal tissue (*n* = 5). **(B)** GABA content in serum (*n* = 10) and whole hippocampal tissue (*n* = 5). **(C)** Representative images of hippocampal histopathological changes (HE staining) in CA1, CA3, and DG subregions. Scale bars: 100 μm. **(D)** Nissl staining revealed changes in Nissl bodies in CA1, CA3, and DG subregions. **(E)** Quantitative analysis of TUNEL-positive cells was performed. Scale bars: 100 μm. **(F)** TUNEL staining displayed neuronal apoptosis in CA1, CA3, and DG. Scale bars: 100 μm. All data are presented as mean ± SEM. For **(A, B)**, data were analyzed by Student’s *t*-test. For **(E, F)**, data were analyzed by two−way ANOVA followed by Tukey’s *post hoc* test. ^*^p < 0.05, ^**^p < 0.01, ^***^p < 0.001.

Analysis of the hippocampal region revealed severe disruptions at both the neurochemical and structural levels. Although no significant changes were observed in the serum levels of Glu or GABA, all of the Model group hippocampal tissue exhibited severe disruption of neurotransmitter homeostasis, marked by a significant elevation in Glu concentration and a concurrent reduction in GABA levels (**Figures 3A, B**). Additionally, the HE staining revealed notable neuronal disorganization, nuclear pyknosis, and decreased cellular density in the CA1, CA3, and DG subregions (**Figure 3C**). Nissl staining further more confirmed extensive depletion of Nissl bodies, suggesting compromised metabolic integrity and biosynthetic capacity of neurons (**Figure 3D**). TUNEL staining indicated increased apoptotic activity, with the animals in the Model group exhibiting a significantly larger number of TUNEL−positive neurons compared with the animals in the Control group (**Figures 3E, F**). Collectively, these findings suggest that chronic stress not only disrupts neurotransmitter disruption but also leads to substantial structural deterioration and neuronal apoptosis in the hippocampus.

### 3.3 Proteomic analysis reveals molecular mechanisms in the hippocampus

**Figure 4:**
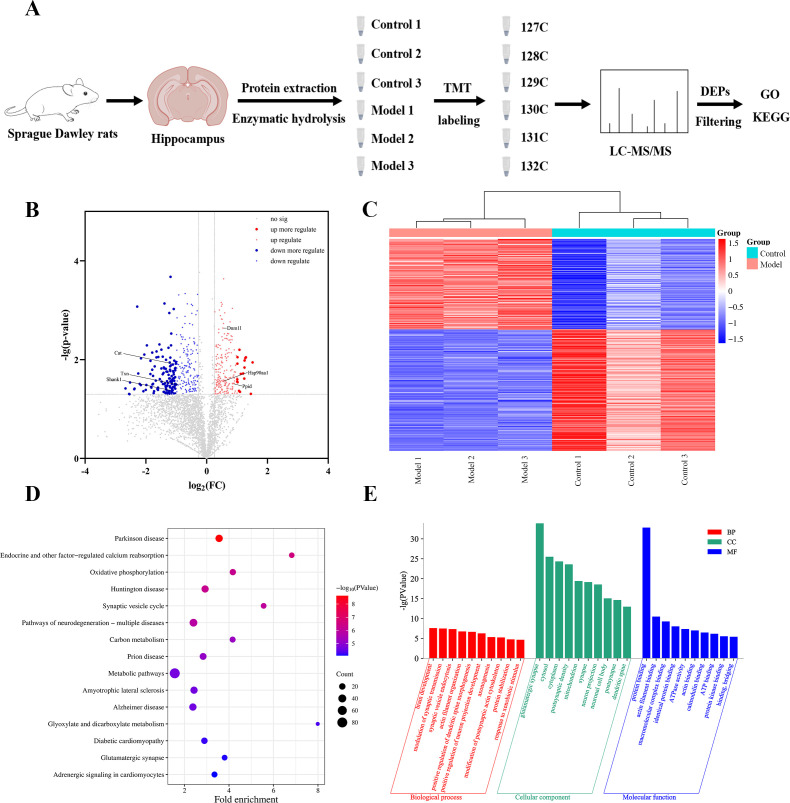
Proteomic analysis identifies molecular mechanisms in the hippocampus. **(A)** Schematic workflow of hippocampal protein profiling using TMT technology. TMT channel assignment: 127C, Control 1; 128C, Control 2; 129C, Control 3; 130C, Model 1; 131C, Model 2; 132C, Model 3. **(B)** Volcano plot displaying DEPs between the Control and Model groups. Significantly upregulated and downregulated proteins (based on fold-change and *p*-value thresholds) are highlighted in red and blue, respectively; non-significant proteins are shown in gray. **(C)** Hierarchical clustering heatmap of DEPs between Control and Model groups (*n* = 3 biological replicates per group). Shades of red represent upregulation, and shades of blue represent downregulation. **(D)** KEGG pathway enrichment analysis of hippocampal DEPs. Dot color indicates the enrichment significance (−log_10_(*p*-value)), with darker blue representing higher significance; dot size reflects the number of proteins enriched in each pathway. **(E)** GO enrichment analysis of DEPs across biological processes, cellular components, and molecular functions.

Comparative proteomic analysis on hippocampal tissues from the control and model groups was conducted using a TMT-labeling approach. Overall, 46,342 peptides and 4,387 proteins were confidently identified. Volcano plot analysis further revealed 212 significantly upregulated (fold change > 1.2, p < 0.05) proteins and 284 downregulated (fold change < 0.83, p < 0.05) proteins (**Figure 4B**). Hierarchical clustering analysis revealed distinct protein expression profiles between the two groups (**Figure 4C**): model-upregulated proteins (Cluster A) were enriched in inflammatory and cytoskeletal signaling pathways, whereas control-enriched proteins (Cluster B) were related to antioxidant defense and metabolism. KEGG pathway enrichment analysis identified 34 significantly enriched pathways, among the Parkinson’s disease pathway was the most prominently enriched. These findings suggest potential involvement of energy metabolism and oxidative stress-related pathways in the observed molecular alterations (**Figure 4D**). GO analysis further indicated enrichment in biological processes related to synaptic function and mitochondrial energy metabolism (**Figure 4E**).

### 3.4 Transcriptomics analysis reveals the underlying hippocampal mechanisms

**Figure 5:**
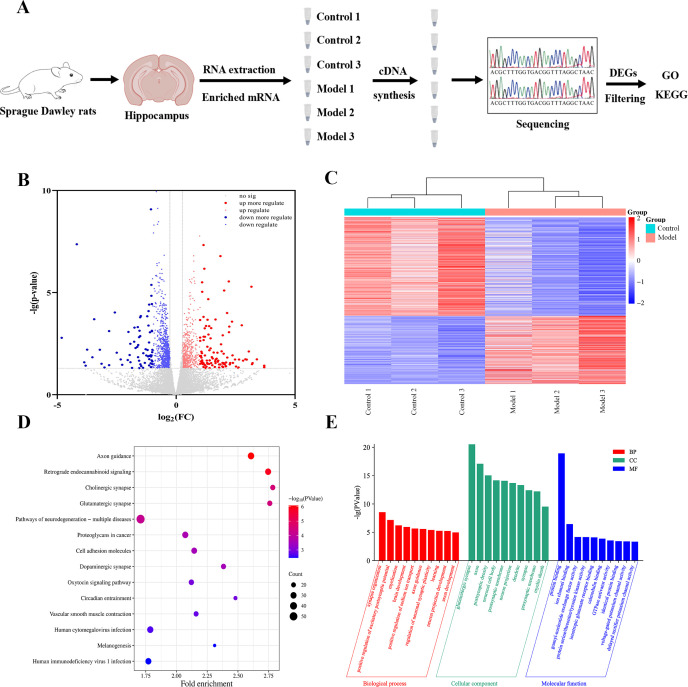
Transcriptomics analysis identifies the underlying hippocampal mechanisms **(A)** Schematic workflow of hippocampal transcriptome profiling using stranded RNA-seq. **(B)** Volcano plot displaying DEPs between the Control and Model groups. Significantly upregulated and downregulated proteins (based on fold-change and *p*-value thresholds) are highlighted in red and blue, respectively; non-significant proteins are shown in gray. **(C)** Hierarchical clustering heatmap of DEPs between Control and Model groups (*n* = 3 biological replicates per group). Shades of red represent upregulation, and shades of blue represent downregulation. **(D)** KEGG pathway enrichment analysis of hippocampal DEPs. Dot color indicates the enrichment significance (−log10(*p*-value)), with darker blue representing higher significance; dot size reflects the number of proteins enriched in each pathway. **(E)** GO enrichment analysis of DEPs across biological processes, cellular components, and molecular functions.

We completed comparative transcriptomic profiling of hippocampal tissues from the Control and Model groups and successfully identified a set of differentially expressed genes (DEGs) (**Figure 5A**). Volcano plot analysis revealed 192 significant DEGs, including 97 upregulated and 95 downregulated genes. Upregulated DEGs were linked to inflammation, while downregulated DEGs were associated with synaptic dysfunction (**Figure 5B**). A heatmap visualization confirmed distinct inter-group expression profiles, with coordinated upregulation of 106 genes in the Model group (red clusters) versus 566 genes enriched in controls (blue clusters) (**Figure 5C**). KEGG identified 63 significantly enriched pathways, with the axon guidance ranking as the most enriched; endocannabinoid, cholinergic, Gluergic, and GABAergic signaling were also enriched, which underscores their pivotal role in synaptic transmission (**Figure 5D**). GO analysis further emphasized dysregulation of synaptic signaling and neuronal development (**Figure 5E**).

### 3.5 Integrated proteomic and transcriptomic analysis identifies coordinated dysregulation of the hippocampal mitophagy pathway

**Figure 6:**
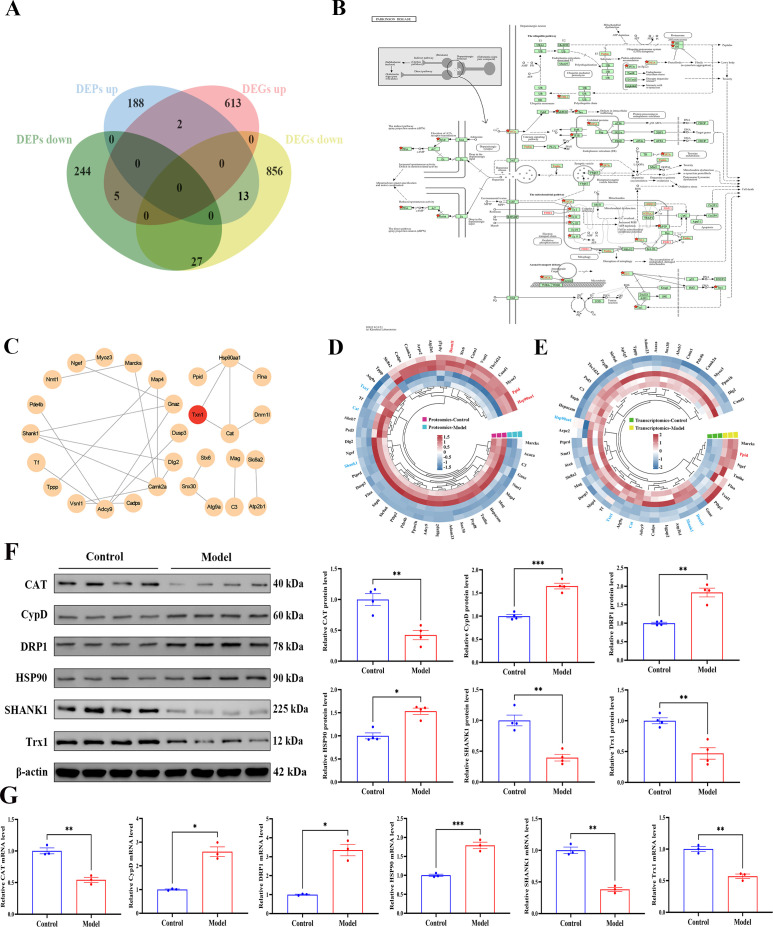
Integrated proteomic and transcriptomic analysis identifies coordinated dysregulation of the hippocampal mitophagy pathway in a rat model of anxious depression. **(A)** Venn diagram displaying the overlap between differentially expressed transcripts and proteins, highlighting molecules with concordant dysregulation at both omics levels. **(B)** KEGG pathway enrichment visualization for Parkinson’s disease, illustrating key genes/proteins involved in mitophagy and neurodegenerative processes. **(C)** PPI network analysis of hippocampal proteins, emphasizing potential antidepressant targets within the mitophagy-immune network. **(D)** Heatmap of DEPs showing expression patterns between Control and Model group. **(E)** Heatmap of DEGs illustrating transcriptional changes under the same conditions. **(F)** Western blot quantification of hippocampal mitophagy-related proteins is presented as representative immunoblots with protein levels normalized to β-actin. **(G)** The qPCR analysis of hippocampal mRNA expression levels. All data are presented as mean ± SEM (*n* = 15 per group). Student’s *t*-test was used for statistical analysis. ^*^p < 0.05, ^**^p < 0.01, ^***^p < 0.001.

Integrated transcriptomic and proteomic analysis revealed coordinated molecular alterations in the hippocampus of model rats. A total of 613 genes were synchronously downregulated at both the mRNA and protein levels (**Figure 6A**). KEGG pathway enrichment analysis identified significant enrichment in mitophagy pathways, accompanied by dysregulation of key genes such as PINK1 and Parkin, suggesting impaired mitochondrial quality control (**Figure 6B**). PPI network analysis further more identified thioredoxin-1 (Trx1 also known as Txn1) as a highly interconnected hub, supporting its purported role as a key mediator bridging oxidative stress and synaptic dysfunction (33) (**Figure 6C**). Both proteomic and transcriptomic profiling confirmed the disrupted expression of molecules related to mitochondrial fission, antioxidant response, and synaptic integrity (**Figures 6D, E**). These findings were further supported by Western blot and qPCR validation (**Figures 6F, G**). Overall, these results suggest that mitochondrial dysfunction and redox imbalance may be associated with synaptic alterations in this model.

### 3.6 CRS/CORT suppresses mitophagy, thereby disrupting damaged mitochondria clearance

**Figure 7:**
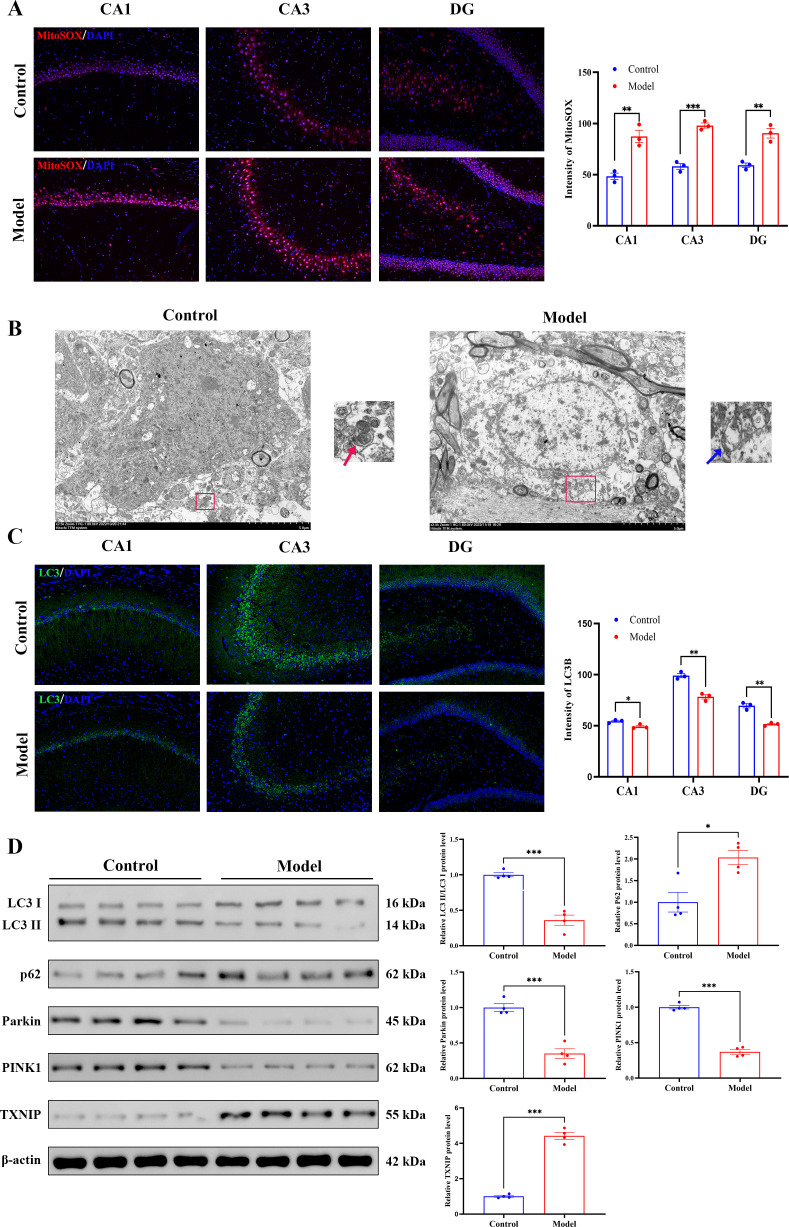
CRS/CORT suppresses the mitophagy, thereby disrupting damaged mitochondria clearance. **(A)** The mtROS levels were assessed using MitoSOX staining (red) with DAPI (blue) as nuclear counterstain. The graph shows quantified fluorescence intensity in the hippocampal subfields. Scale bars: 100 μm. **(B)** Ultrastructural analysis by TEM: intact mitochondria (red arrow, Control group) and mitochondria with damaged cristae (blue arrow, Model group). Scale bars: 5 μm. **(C)** Autophagosome formation was visualized via LC3B immunofluorescence (green) and DAPI (blue). The graph displays quantified LC3B intensity across hippocampal subregions. Scale bars: 100 μm. **(D)** Western blot analysis of mitophagy-related proteins, including the LC3-II/I ratio and expression levels of P62, Parkin, PINK1, and TXNIP, normalized to β-actin. All data are presented as mean ± SEM (*n* = 3 per group). For **(A, C)**, data were analyzed by two−way ANOVA followed by Tukey’s *post hoc* test. For **(D)**, data were analyzed by Student’s *t*-test. ^*^p < 0.05, ^**^p < 0.01, ^***^p < 0.001.

Functional assessment of mitochondrial status revealed significant pathological alterations. MitoSOX Red fluorescence staining demonstrated a marked increase in superoxide fluorescence intensity in hippocampal slices from animals in the Model group; this finding indicates excessive mitochondrial superoxide production (**Figure 7A**). Ultrastructural examination by TEM revealed mitochondrial swelling, characterized by fragmented cristae and occasional rupture of the outer membrane, indicative of severe structural damage (**Figure 7B**). Immunofluorescence analysis showed a significant reduction in LC3B-positive puncta, which spatially colocalized with areas of elevated mtROS (**Figure 7C**). Western blot analysis demonstrated a decreased LC3-II/LC3-I ratio, reduced expression levels of PINK1 and Parkin, the accumulation of p62, and significant upregulation of TXNIP (**Figure 7D**). Collectively, these findings indicate that chronic stress disrupts the clearance of damaged mitochondria by compromising the PINK1/Parkin-mediated mitophagy pathway.

### 3.7 Mito-TEMPO alleviates anxiety and depression-like behaviors

Intervention with the mitochondria-targeted antioxidant Mito-TEMPO, administered during the stress paradigm, significantly alleviated the observed behavioral abnormalities. In the OFT, the animals in the Model group exhibited significant reductions in both total distance traveled and central distance compared with the animals in the Ccontrol group; these deficits were markedly attenuated by Mito-TEMPO treatment (**Figures 2B, C, H**). Similarly, in the EPM test, Mito-TEMPO significantly increased the OE% and OT% the open arms compared with the animals in the Model group (**Figures 2D, E, I**). Furthermore, Mito-TEMPO intervention reversed anhedonic behavior, as evidenced by normalized sucrose preference in the SPT, and attenuated behavioral despair, demonstrated by reduced immobility time in the FST (**Figures 2F, G**). Collectively, these findings demonstrate that pharmacological targeting of mtROS is sufficient to comprehensively reverse the CRS/CORT-induced behavioral phenotype.

### 3.8 Mito-TEMPO protects against mitochondrial damage and restores mitophagy

**Figure 8:**
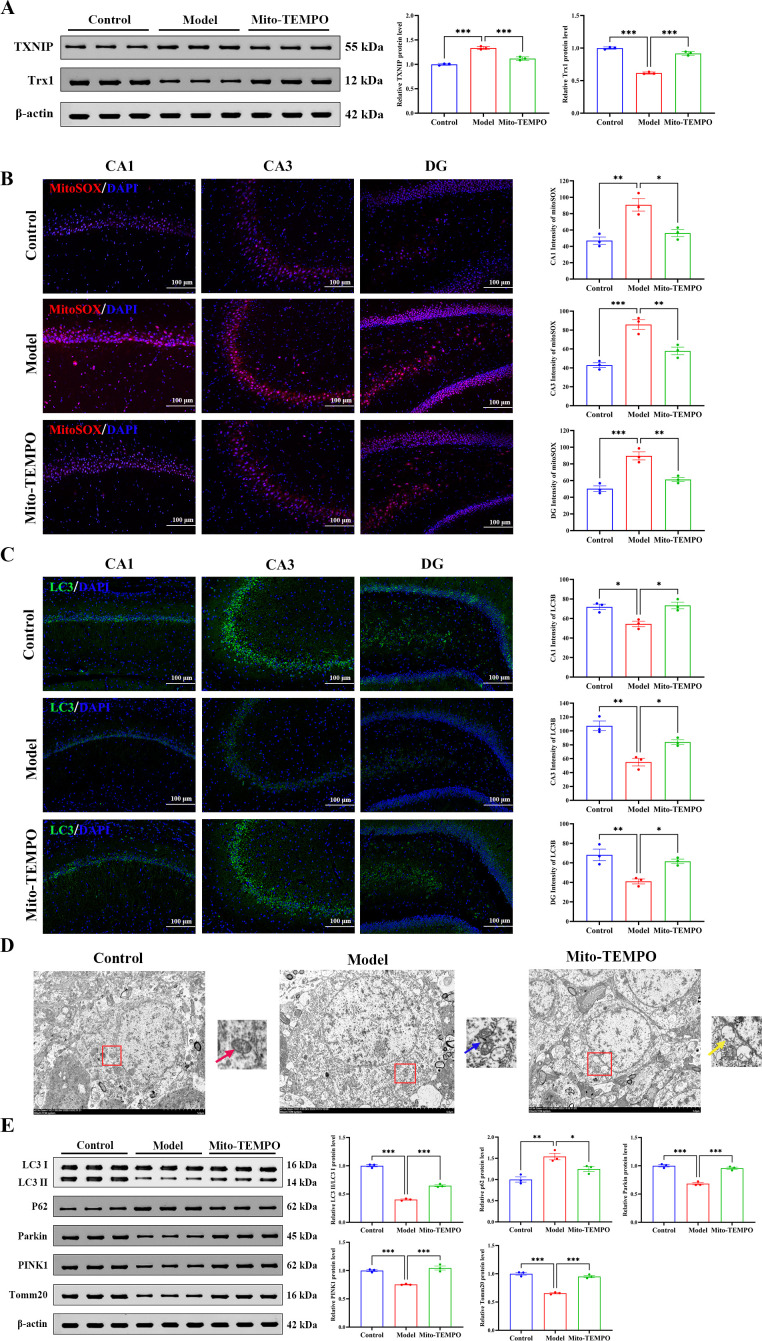
Mito-TEMPO enhances mitophagy and attenuates mitochondrial damage. **(A)** Protein expression of TXNIP and Trx1 in the hippocampus: representative immunoblots and quantitative analysis normalized to β-actin. **(B)** Mitochondrial ROS levels visualized by MitoSOX staining (red) with DAPI nuclear counterstaining (blue). Graph shows quantified fluorescence intensity across hippocampal subregions. Scale bars: 100 μm. **(C)** Autophagosome formation detected via LC3 immunofluorescence (green) and DAPI (blue). Quantified LC3 puncta intensity is presented. Scale bars: 100 μm. **(D)** Ultrastructural morphology of mitochondria observed by TEM: intact cristae in control (red arrow), disrupted cristae and outer membrane rupture in model (blue arrow), and autophagosome engulfing damaged mitochondrion in Mito-TEMPO group (yellow arrow). Scale bars: 5 μm. **(E)** Western blot analysis of mitophagy-related proteins of LC3-II/I ratio, p62, Parkin, PINK1, and Tomm20, normalized to β-actin. All data are presented as mean ± SEM (*n* = 3 per group). One-way ANOVA with Tukey’s *post hoc* test was used for statistical analysis. ^*^p < 0.05, ^**^p < 0.01, ^***^p < 0.001.

Mito-TEMPO prevented mitochondrial damage and restored mitophagy, concomitant with the effective reversal of stress-induced Trx1 downregulation and attenuation of TXNIP upregulation, as determined by Western blot analysis (**Figure 8A**). MitoSOX Red staining revealed a robust surge in mitochondrial superoxide fluorescence in the animals in the Model group, an effect almost completely quenched by Mito-TEMPO. This finding corroborates the relief of mitochondrial oxidative stress (**Figure 8B**). Furthermore, immunofluorescence analysis revealed diminished LC3 intensity and fewer LC3B-positive puncta, with both restored by Mito-TEMPO administration (**Figure 8C**). TEM imaging revealed the preservation of mitochondrial ultrastructure, including intact cristae and an elevated abundance of mitophagosomes (**Figure 8D**). Western blot analysis furthermore confirmed the functional rescue of mitophagy, showing an increased LC3-II/LC3-I ratio; elevated protein levels of Parkin, PINK1, and the mitochondrial outer membrane protein Tomm20; and decreased p62 accumulation (**Figure 8E**).

### 3.9 Mito-TEMPO suppresses the activation of the NLRP3 inflammasome pathway

**Figure 9:**
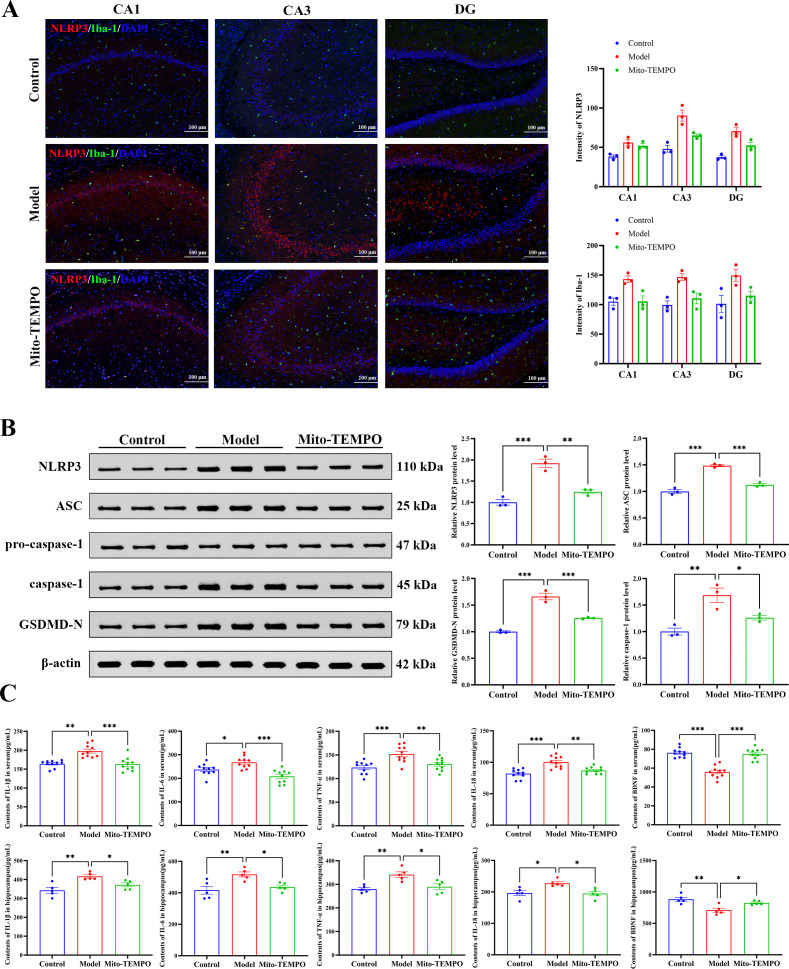
Mito-TEMPO attenuates inflammatory responses and NLRP3 inflammasome activation. **(A)** Representative immunofluorescence images of NLRP3 (red) and Iba-1 (green) in the hippocampus, with DAPI nuclear staining (blue). Quantified fluorescence intensity of NLRP3 and Iba-1 in hippocampus subregions (*n* = 3). Scale bars: 100 μm. **(B)** Protein expression levels of NLRP3 inflammasome components (ASC, pro-caspase-1, caspase-1, GSDMD-N) in hippocampal tissue. Quantitative immunoblot results normalized to β-actin and expressed relative to the control group (*n* = 3). **(C)** Effects of Mito-TEMPO on concentrations of IL-1β, IL-6, TNF-α, IL-18, and BDNF in serum (*n* = 10) and the hippocampus (*n* = 5). All data are presented as mean ± SEM. For **(A)**, data were analyzed by two−way ANOVA followed by Tukey’s *post hoc* test. For **(B, C)**, data were analyzed by one−way ANOVA followed by Tukey’s *post hoc* test. ^*^p < 0.05, ^**^p < 0.01, ^***^p < 0.001.

We also systematically evaluated the anti-inflammatory effects of Mito-TEMPO were systematically evaluated. Immunofluorescence co-staining demonstrated that Mito-TEMPO treatment significantly reduced the stress-induced co-localization and fluorescence intensity of NLRP3 and the microglial marker Iba-1 in hippocampal regions (**Figure 9A**). Western blot analysis further more confirmed that chronic stress significantly up-regulated the protein levels of key components in the NLRP3 inflammasome pathway, including NLRP3, ASC, caspase-1, and the pyroptosis executioner protein GSDMD-N (**Figure 9B**). Concurrent ELISA results demonstrated that chronic stress led to an elevation of pro-inflammatory cytokines (IL-1β, IL-18, IL-6, TNF-α) and a reduction in BDNF levels in both hippocampal tissues and serum samples. Importantly, Mito-TEMPO administration effectively reversed all these molecular alterations (**Figure 9C**). These findings indicate that Mito-TEMPO exerts broad anti-inflammatory effects by inhibiting the NLRP3 inflammasome pathway and modulating cytokine networks.

### 3.10 Mito-TEMPO exerts neuroprotection by restoring neuronal morphology and inhibiting apoptosis

**Figure 10:**
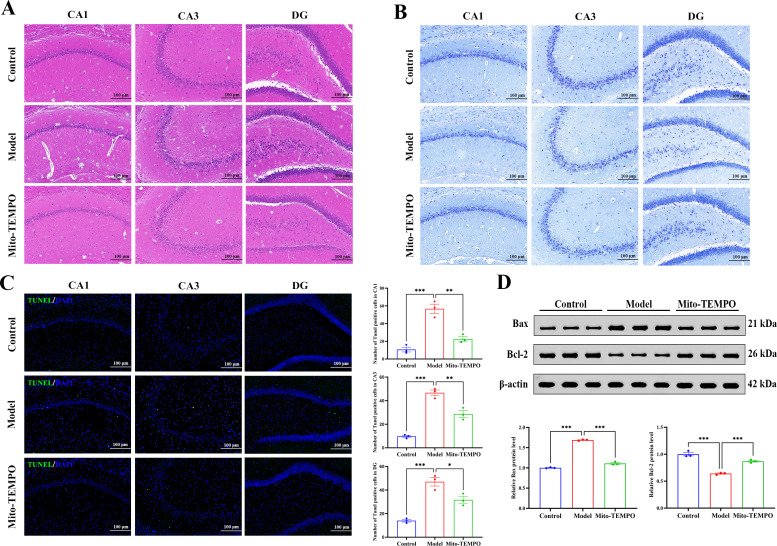
Mito-TEMPO attenuates histopathological damage and neuronal apoptosis in the hippocampus. **(A)** Representative images of HE staining in the hippocampus subregions. Scale bars: 100 μm. **(B)** Nissl staining illustrating neuronal architecture and Nissl body integrity in the hippocampus subregions. Scale bars: 100 μm. **(C)** TUNEL staining was used to detect apoptotic neurons in the hippocampal subregions. (Left panel) Quantitative analysis of TUNEL-positive cells. Scale bars: 100 μm. **(D)** Western blot analysis of apoptosis-related proteins Bax and Bcl-2. Protein expression levels were normalized to β-actin. All data are presented as mean ± SEM (*n* = 3 per group). One-way ANOVA with Tukey’s *post hoc* test was used for statistical analysis. ^*^p < 0.05, ^**^p < 0.01, ^***^p < 0.001.

Mito-TEMPO exhibited robust neuroprotective efficacy in the hippocampus. We found that HE and Nissl staining revealed attenuated neuronal disorganization, reduced nuclear pyknosis, and restored Nissl body density in hippocampal subfields (**Figures 10A, B**). Concordantly, TUNEL staining demonstrated a marked reduction in the number of apoptotic neurons (**Figure 10C**). Mechanistically, Western blot analysis confirmed that Mito-TEMPO normalized the Bax/Bcl-2 ratio, thereby shifting the apoptotic balance toward cell survival (**Figure 10D**). Collectively, these results support the idea that mtROS scavenging not only rectifies functional and inflammatory perturbations but also confers structural and cellular resilience in the stressed hippocampus; our findings highlight the therapeutic potential of mitochondrial-targeted interventions in anxious depression.

### 3.11 Knockdown of TXNIP in microglia attenuates LPS-induced inflammasome activation and protects neurons

**Figure 11:**
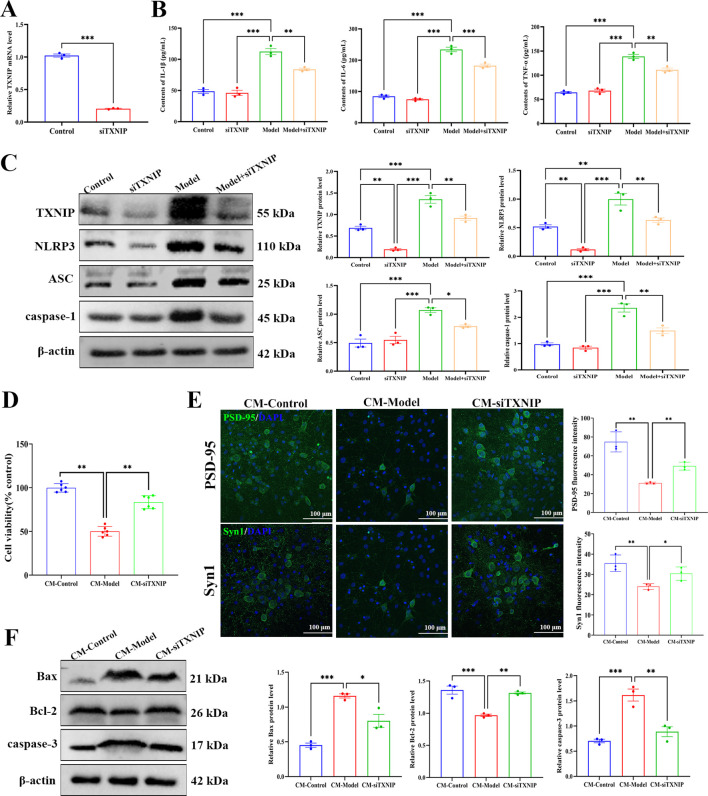
Knockdown of TXNIP in BV2 microglia attenuates LPS-induced neuroinflammation and subsequent HT22 neuronal injury. **(A)** TXNIP mRNA levels in BV2 cells after TXNIP siRNA transfection. **(B)** Concentrations of proinflammatory cytokines (IL-1β, IL-6, TNF-α) in BV2 cell supernatants, measured by ELISA. **(C)** Western blot analysis of TXNIP, NLRP3, ASC, and caspase-1 protein expression in BV2 cells, with β-actin as the loading control. **(D)** Viability of HT22 neurons treated with conditioned medium (CM) from differently treated BV2 cells, assessed by CCK-8 assay. **(E)** Immunofluorescence staining of PSD-95, Syn1 in HT22 cells (scale bar = 100 μm), with quantitative fluorescence intensity analysis. **(F)** Western blot detection of Bax, Bcl-2, cleaved caspase-3 in HT22 cells, with β-actin as the loading control. All data are presented as mean ± SEM (*n* = 3 per group). For **(A)**, data were analyzed by Student’s *t*-test. One-way ANOVA with Tukey’s *post hoc* test was used for other statistical analysis. ^*^p < 0.05, ^**^p < 0.01, ^***^p < 0.001.

To investigate the causal role of TXNIP in microglial NLRP3 inflammasome activation and its impact on neuronal injury, TXNIP knockdown was performed in BV2 cells followed by conditioned medium (CM) transfer to HT22 neurons. qPCR analysis confirmed efficient TXNIP silencing in siRNA-transfected BV2 cells (**Figure 11A**). LPS stimulation markedly induced the secretion of pro-inflammatory cytokines (IL-1β, IL-6, TNF-α), which was significantly abrogated by TXNIP knockdown (**Figure 11B**). Consistently, Western blot analysis revealed that TXNIP suppression inhibited LPS-upregulated expression of NLRP3, ASC, and caspase-1 (**Figure 11C**), demonstrating TXNIP’s regulation of the NLRP3 inflammasome.

When HT22 neurons were treated with CM from differentially treated BV2 cells, CCK-8 assay showed that CM-Model reduced HT22 viability, an effect reversed by TXNIP knockdown (**Figure 11D**). Moreover, CM-Model induced neuronal injury and decreased the immunofluorescence intensity of synaptic markers PSD-95 and Syn1, and increased the expression of pro-apoptotic proteins Bax and cleaved caspase-3 while decreasing anti-apoptotic Bcl-2 in HT22 cells, with all these changes mitigated by TXNIP knockdown (**Figures 11E, F**). Collectively, these results identify TXNIP as a key molecular link connecting microglial inflammatory activation to neuronal dysfunction and apoptosis, highlighting the pathological significance of the TXNIP/NLRP3 axis in stress-induced neuronal injury.

## 4 Discussion

Anxious depression is a complex neuropsychiatric disorder characterized by a close link between chronic inflammatory responses and oxidative stress in its pathogenesis and progression (34). ROS, predominantly produced in mitochondria, not only act as mediators of cellular damage but also play a critical role as signaling molecules involved in regulating inflammation and neuronal survival (35). Excessive ROS activates the microglial inflammasome, triggering the release of pro-inflammatory cytokines (e.g., TNF-α, IL-1β, and IFN-γ). These cytokines further induce ROS production, forming a vicious cycle that contributes to depression (36). This study, using a CRS/CORT model that induces robust anxiety- and depression-like behaviors in rats, unveils mitochondrial dysfunction and neuroinflammation as central pathological mechanisms underlying anxious depression phenotypes.

We conducted integrated proteomic and transcriptomic analyses of the hippocampus. In both datasets, convergent alterations were identified in key pathways related to mitochondrial function, oxidative stress, and synaptic integrity, suggesting that these processes are key components of the pathological response to chronic stress. These disruptions were marked by reduced antioxidant defense and elevated pro-inflammatory signaling. Bioinformatics enrichment linked these changes to excitatory synaptic dysfunction and neuroinflammation. The concurrent dysregulation of synaptic and inflammatory genes suggests a vicious cycle: mitochondria-derived oxidative stress drives synaptic impairment and inflammation, which in turn exacerbates neuronal damage. However, these relationships are inferred from associative analyses rather than directly demonstrated causal links.

Studies have shown that mitochondrial dysfunction and the subsequent accumulation of mitochondrial reactive oxygen species serve as the basis for NLRP3 inflammasome activation and the onset of inflammatory responses (37). However, selective mitophagy can promptly recognize and eliminate dysfunctional mitochondria, thereby maintaining mitochondrial network homeostasis. Under chronic stress conditions, this process appears to be impaired, leading to extensive accumulation of damaged mitochondria within cells. Damaged mitochondria themselves activate multiple inflammatory responses by releasing a series of damage-associated molecular patterns, among which mtROS, along with mtDNA, cardiolipin, and ATP, can be recognized by NLRP3 to drive inflammasome assembly and activation (14, 38). KEGG pathway enrichment analysis revealed that the mitophagy pathway was one of the most significantly enriched and severely dysregulated pathways in our dataset. Subsequent Western blot analysis showed a decreased LC3-II/LC3-I ratio, reduced expression levels of PINK1 and Parkin, and aggregation of p62 protein. These findings collectively indicate impaired mitophagy, which may represent an important upstream event linking oxidative stress to neuroinflammatory responses.

**Figure 12:**
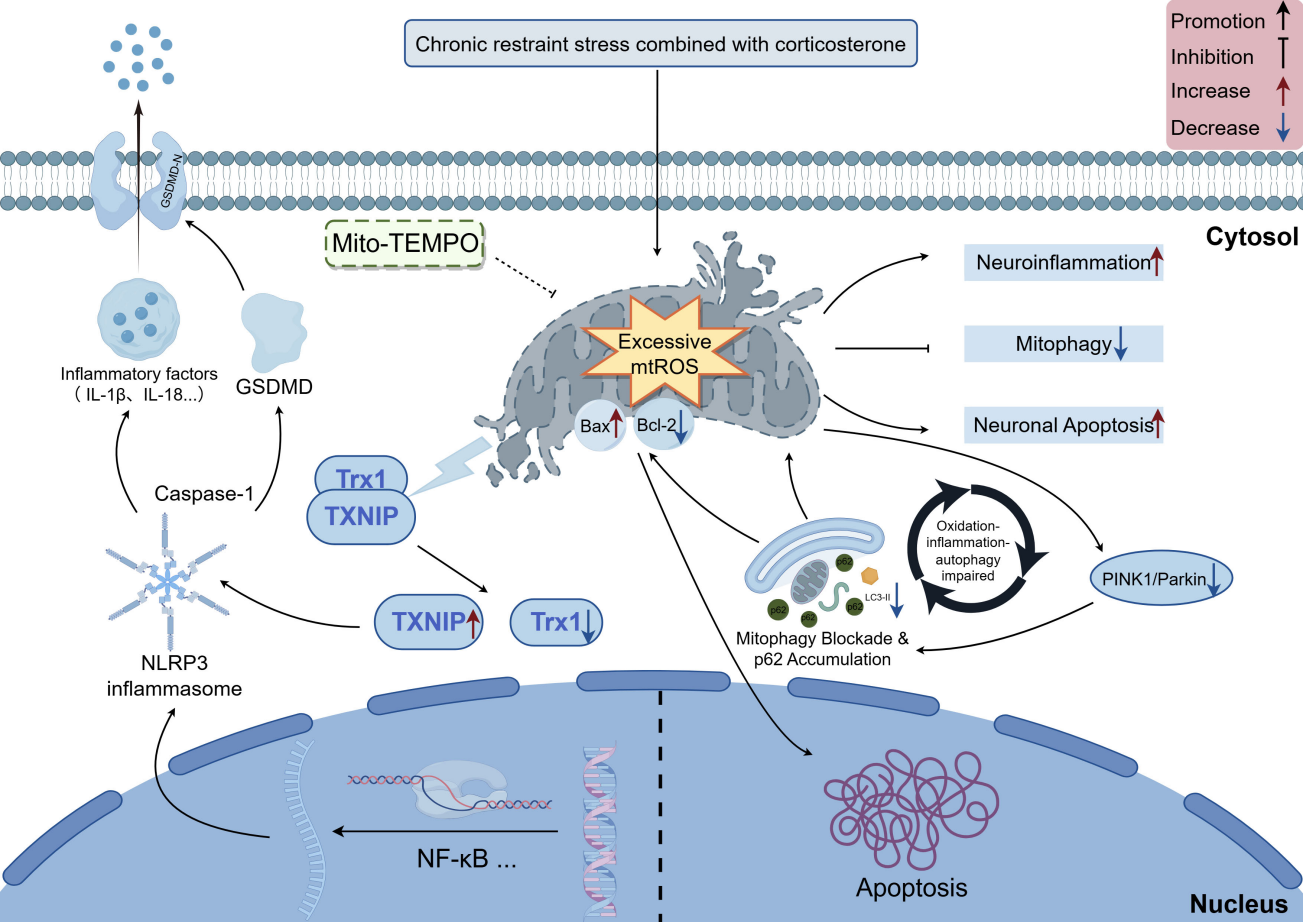
The schematic illustrates that CRS/CORT elicits hippocampal mitochondrial injury and pathological mtROS accumulation, which in turn drives anxious depression by epigenetically regulating TXNIP-mediated NLRP3 inflammasome activation while simultaneously impairing mitophagy.

Building on the complex pathological mechanisms underlying anxious depression, our findings targeted mitochondrial oxidative stress as a key intervention node. To this end, we employed Mito-TEMPO, a mitochondria-targeted antioxidant compound with demonstrated significant antidepressant and antioxidant effects in models of depression (39). Via scavenging, Mito-TEMPO not only inhibited downstream NLRP3 inflammasome activation but also significantly restored mitophagic flux, as evidenced by an increased LC3-II/LC3-I ratio, upregulation of PINK1/Parkin expression, and enhanced mitophagosome formation. These findings are consistent with the results of earlier studies (40). Together, these results support the notion that modulation of mitochondrial oxidative stress may influence both inflammatory activation and mitochondrial quality control processes under stress conditions. As shown in **Figure 12**, mitochondrial oxidative stress may act as a mechanistic link between mitochondrial dysfunction and neuroinflammation in this model.

To further explore the upstream mechanisms linking oxidative stress to inflammation, we focused on TXNIP, a redox-sensitive regulator, and its interaction with Trx1. Excessive mtROS has been reported to promote the dissociation of the Trx1-TXNIP complex, thereby facilitating TXNIP binding to NLRP3 and promoting inflammasome activation (41, 42). TXNIP and Trx1 are functionally antagonistic redox regulatory proteins, their interaction and localization are critically influenced by cellular redox state (43). Trx1 overexpression typically confers protection against oxidative stress and effectively promotes mitochondrial biogenesis (44). In our study, CRS/CORT decreased Trx1 expression and increased TXNIP levels, while Mito-TEMPO treatment reversed these changes. To further examine the functional role of TXNIP, we performed *in vitro* loss-of-function experiments. TXNIP knockdown in BV2 microglial cells significantly attenuated LPS-induced NLRP3 inflammasome activation and reduced the release of pro-inflammatory cytokines. Moreover, conditioned medium derived from TXNIP-silenced BV2 cells markedly alleviated neuronal injury in HT22 cells. These findings provide functional evidence that TXNIP mediates microglia-driven inflammatory responses and contributes to subsequent neuronal damage.

Accumulating evidence has highlighted the significant role of NLRP3 inflammasome activation in the pathogenesis of depression (45). Various stressors, including LPS and CORT, can trigger NLRP3 inflammasome assembly and activation in emotion-related brain regions such as the hippocampus (particularly in microglia), leading to caspase-1-dependent maturation and release of pro-inflammatory cytokines (46, 47). Chronic stress elevates brain ROS levels, downregulates antioxidant enzyme activity, and induces depressive-like behaviors (48). Our immunofluorescence results showed increased co-localization signals of NLRP3 and Iba-1 in the hippocampus following stress exposure, suggesting activation of microglial inflammasomes. This effect, along with the elevated protein levels of pathway components and inflammatory cytokines confirmed by Western blot and ELISA, was suppressed by Mito-TEMPO, consistent with earlier findings (49, 50). Collectively, these findings indicate that mitochondrial oxidative stress is associated with microglia-related inflammatory activation in this model.

The ROS/TXNIP/NLRP3 signaling axis has been proposed as a key redox-sensitive inflammatory pathway in which ROS promote the dissociation of TXNIP from Trx, thereby enabling TXNIP to interact with NLRP3 and initiate inflammasome activation (51). Consistent with this concept, a chronic mild stress model study further demonstrated that UCP2 deficiency exacerbates ROS accumulation and enhances NLRP3 expression and TXNIP-NLRP3 interaction in astrocytes, effects that can be attenuated by ROS inhibition (52). Together with our findings showing mitochondrial oxidative stress–driven activation of the TXNIP/NLRP3 pathway and its modulation by Mito-TEMPO and TXNIP knockdown, these results collectively support that this signaling axis serves as a central molecular bridge linking oxidative stress to neuroinflammatory responses under chronic stress conditions.

This investigation has several limitations. First, the omics analyses were designed as exploratory and hypothesis-generating approaches. The relatively small sample size and the use of permissive statistical thresholds without FDR correction may limit the robustness of the identified molecular signatures; therefore, these findings should be interpreted with caution as preliminary evidence. Second, although we combined *in vivo* pharmacological intervention and *in vitro* loss-of-function experiments to support the functional relevance of key molecules, certain mechanistic interactions within the ROS/TXNIP/NLRP3 signaling pathway still require further direct validation. Finally, the CRS/CORT model induces stress-related behavioral phenotypes resembling anxiety and depression but does not fully recapitulate the complexity of human anxious depression, and therefore the findings should be interpreted within a preclinical context.

## 5 Conclusion

This study demonstrates that the ROS/TXNIP/NLRP3 signaling pathway serves as the central molecular mediator of the crosstalk between mitochondrial oxidative stress and neuroinflammation, representing a core pathogenic mechanism in anxious depression. Specifically, excessive mtROS disrupts the Trx1/TXNIP redox balance, promotes TXNIP-NLRP3 binding to activate the inflammasome, and suppresses PINK1/Parkin-mediated mitophagy, which together exacerbate neuronal damage and anxiety- and depression-like behavioral abnormalities, findings supported by evidence that targeting TXNIP in microglia attenuates inflammasome activation and protects neurons from inflammatory injury.

## Data Availability

The datasets presented in this study can be found in online repositories. The names of the repository/repositories and accession number(s) can be found in the article/**Supplementary Material**.
