# Supplementary material for: From mitochondrial oxidative stress to neuroinflammation: integrated proteomic and transcriptomic profiling reveals the role of the ROS/TXNIP/NLRP3 signaling pathway in anxious depression
Source: Front Immunol. 2026 May 19;17:1803956. doi: 10.3389/fimmu.2026.1803956 (PMC13226016; doi:10.3389/fimmu.2026.1803956)
Supplement: Supplementary file 1 [file Table1.docx]

Table S1. Primers and siRNA sequences used in this study

Part A: qPCR primers

| Gene | Forward (5'→3') | Reverse (5'→3') |
| --- | --- | --- |
| CAT | GCAGATACCTGTGAACTGTC | GTAGAATGTCCGCACCTGAG |
| CypD | CACCTTCCACAGGGTGATCC | AGCAACAGTGTAGCGCAATG |
| DRP1 | GAGAACTACCTTCCGCTGTATCGC | CACCATCTCCAATTCCACCACCTG |
| HSP90 | TGGACAGCAAACATGGAGAG | AGACAGGAGCGCAGTTTCAT |
| SHANK1 | GGAGCAGCAGCAACTACTTC | GCCATCTTCTTCATCTCCTG |
| Trx1 | GTGAAGCAGATCGAGAGCAAG | CGTGGCTGAGAAGTCAACTACTA |
| TXNIP | GACGATGTGGACGACTCTCAAGAC | GTTGTTGTTAAGGACGCACGGATC |
| β-actin | TTCCTTCCTGGGTATGGAAT | GAGGAGCAATGATCTTGATC |

Part B: siRNA sequences targeting mouse TXNIP

| siRNA Name | Sequence (5' to 3') |
| --- | --- |
| siTXNIP-1062 (Sense) | CGAUUGUGACGACUCUCAAGATT |
| siTXNIP-1062 (Antisense) | UCUUGAGAGUCGUCACAAUCGTT |

**Table S2** Summary of all primary antibodies for Western blot

| Primary antibodies | Type | Source | WB | IF |
| --- | --- | --- | --- | --- |
| HSP90 | Rabbit poly | Proteintech (13171-1-AP) | 1/3000 |  |
| CYPD | Rabbit poly | Proteintech (12716-1-AP) | 1/1000 |  |
| SHANK1 | Mouse mono | Abcam (ab94576) | 2ug/ml |  |
| CAT | Rabbit poly | Proteintech (21260-1-AP) | 1/5000 |  |
| DRP1 | Rabbit poly | Proteintech (12957-1-AP) | 1/1000 |  |
| Trx1 | Rabbit poly | Proteintech (14999-1-AP) | 1/5000 |  |
| LC3B | Rabbit poly | Proteintech (14600-1-AP) | 1/2000 | 1:400 |
| TXNIP | Rabbit poly | Proteintech (18243-1-AP) | 1/1000 |  |
| P62 | Mouse mono | Abcam (ab10912) | 1/10000 |  |
| Parkin | Rabbit poly | Proteintech (14060-1-AP) | 1/2000 |  |
| PINK1 | Rabbit poly | Proteintech (23274-1-AP) | 1/1000 |  |
| Tomm20 | Rabbit poly | Proteintech (11802-1-AP) | 1/1000 |  |
| NLRP3 | Rabbit poly | Proteintech (19771-1-AP) | 1/1000 | 1:200 |
| ASC | Rabbit poly | Proteintech (10500-1-AP) | 1/5000 |  |
| Pro-caspase-1 | Rabbit mono | Abcam (Ab179515) | 1/1000 |  |
| Caspase-1 | Rabbit poly | Proteintech (22915-1-AP) | 1/2000 |  |
| Caspase-3 | Rabbit poly | Proteintech  （19677-1-AP） | 1/2000 |  |
| GSDMD-N | Rabbit poly | Proteintech (20770-1-AP) | 1/2000 |  |
| Bax | Rabbit poly | Proteintech (50599-2-Ig) | 1/2000 |  |
| Bcl-2 | Rabbit poly | Proteintech (26593-1-AP) | 1/2000 |  |
| GAPDH | Mouse mono | Proteintech (60004-1-Ig) | 1/5000 |  |
| β-actin | Mouse mono | Proteintech (66009-1-Ig) | 1/5000 |  |
| Iba-1 | Rabbit mono | Abcam  ab178846 |  | 1:1000 |
